# Complete Genome and Partial Megaplasmid Sequences of Mycobacterium pseudoshottsii Strain NJB1907-Z4, Isolated from an Aquarium-Reared Japanese Sardine (Sardinops melanostictus) in Japan

**DOI:** 10.1128/mra.00785-22

**Published:** 2022-11-09

**Authors:** Takeshi Komine, Hanako Fukano, Mitsunori Yoshida, Mari Inohana, Yoshihiko Hoshino, Osamu Kurata, Shinpei Wada

**Affiliations:** a Laboratory of Aquatic Medicine, School of Veterinary Medicine, Nippon Veterinary and Life Science University, Musashino, Tokyo, Japan; b Department of Mycobacteriology, Leprosy Research Center, National Institute of Infectious Diseases, Higashi-Murayama, Tokyo, Japan; Montana State University

## Abstract

Mycobacterium pseudoshottsii, a slow-growing nontuberculous mycobacterium, has been isolated from wild and cultured fish. We report here the complete genome and partial megaplasmid sequences of a strain isolated from an aquarium-reared Japanese sardine (Sardinops melanostictus) in Japan, *M. pseudoshottsii* NJB1907-Z4.

## ANNOUNCEMENT

Mycobacterium pseudoshottsii, a slow-growing nontuberculous mycobacterium that can cause mycobacteriosis in fish (1), has been reported in wild and cultured fish (2–5). This pathogen produces a macrolide toxin, mycolactone, and is classified as a mycolactone-producing mycobacterium (6).

A Japanese sardine (Sardinops melanostictus) was randomly sampled from a tank at the Tokyo Sea Life Park in which mycobacteriosis occurred in 2019. The liver was collected from the fish and subjected to mycobacterial culture, resulting in the isolation of *M. pseudoshottsii* strain NJB1907-Z4 (7). After the strain was streaked onto two 2% Ogawa slants (Kyokuto Pharmaceutical Industrial Co., Ltd., Japan) and cultivated for 4 weeks at 25°C, DNA was extracted from colonies on each slant. DNA from one slant was sequenced using the Illumina HiSeq X platform and from the other using the PacBio Sequel platform.

Genomic DNA for Illumina sequencing was extracted from approximately 1.1 × 10^9^ CFU of NJB1907-Z4 (8). The Illumina library was prepared using the Nextera XT DNA library preparation kit (Illumina, USA) and sequenced on the Illumina HiSeq X instrument (2 × 150-bp format). The raw read quality was assessed using FastQC v0.11.9 (9). The Illumina reads were trimmed for quality using fastp v0.20.1 (10), yielding 5,474,482 sequences.

High-molecular-weight (HMW) DNA for the PacBio Sequel platform was extracted from 2.2 × 10^9^ CFU of the strain with acid-washed glass beads (G8772; Sigma, USA) using NucleoBond HMW DNA (Macherey-Nagel GmbH & Co. KG, Germany). Libraries were prepared using the SMRTbell Express template prep kit 2.0 (Pacific Biosciences, USA) and sequenced on the PacBio Sequel platform. The PacBio raw reads were converted using bam2fastq (https://github.com/jts/bam2fastq) and quality filtered using Filtlong v0.2.1 (https://github.com/rrwick/Filtlong) with the trimmed Illumina reads. Using Filtlong, 64,108 sequences were selected, with an average length of 7,799 bp and an *N*_50_ value of 8,152 bp.

*De novo* assembly of the PacBio reads was performed using Flye v2.9+galaxy0 (11). The trimmed Illumina and PacBio reads were mapped to the PacBio assembly using the Burrows-Wheeler Aligner v0.7.17 (r1188) (12) and SAMtools v1.15.1 (13) for polishing (three rounds) with Pilon v1.24 (14). A 6,051,062-bp circular chromosomal contig was generated, with a GC content of 65.6% and an average coverage of 189×. A linear plasmid of 117,040 bp was also generated, with a GC content of 62.9% and an average coverage of 630×. Default settings were used for all software.

Annotation was performed using the DDBJ FAST Annotation and Submission Tool v1.2.16 (https://dfast.nig.ac.jp). The chromosome contained 5,617 genes, including 5,563 coding DNA sequences, 3 rRNAs, 50 tRNAs, and 1 CRISPR; the plasmid contained 103 coding DNA sequences.

**FIG 1:**
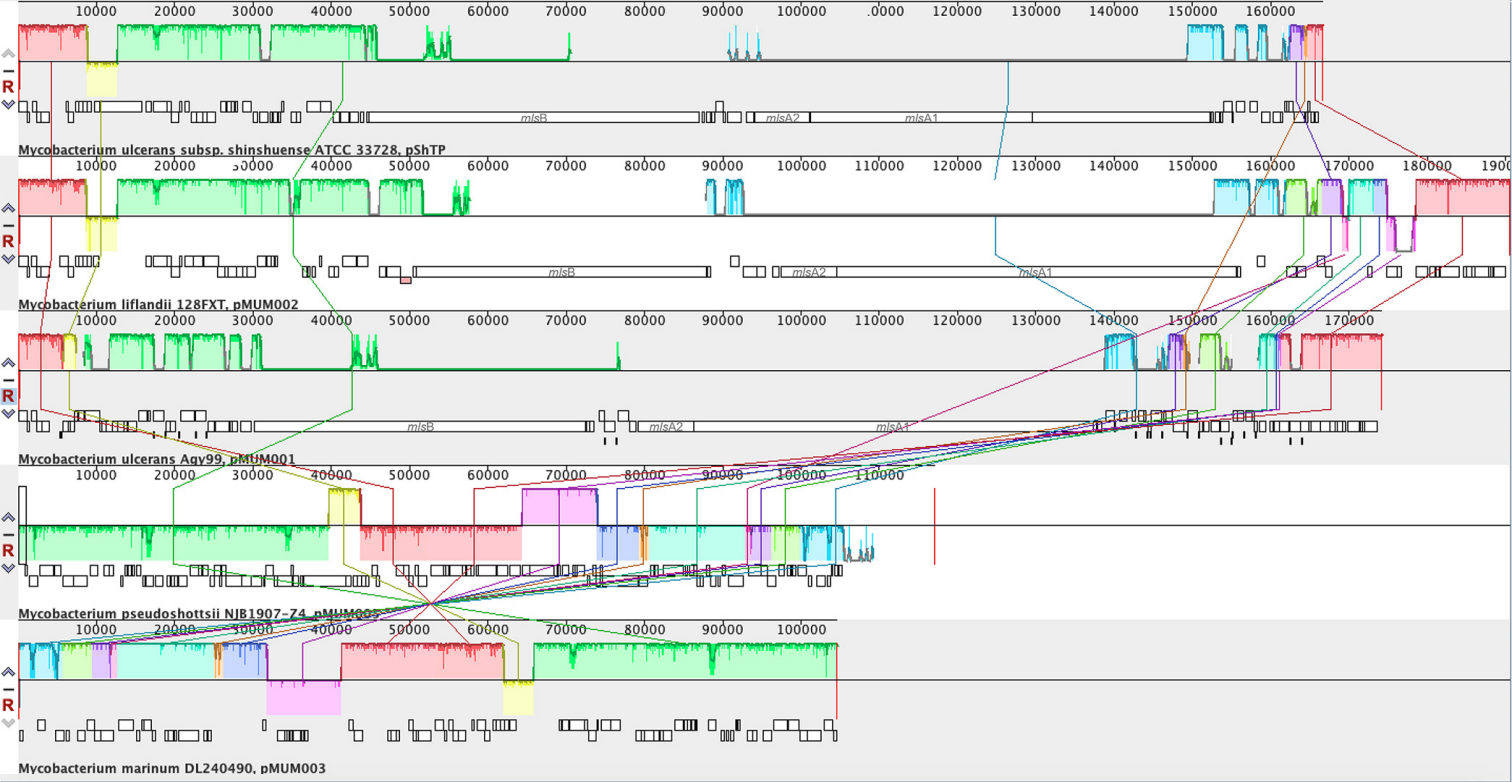
MAUVE alignment of plasmids (top to bottom, pShTP, pMUM002, pMUM001, pMUM005, and pMUM003) of mycolactone-producing mycobacteria.

Average nucleotide identity values of 98.5% to 99.8% between the plasmid and those of other mycolactone-producing mycobacteria from the National Center for Biotechnology Information GenBank database (Mycobacterium ulcerans, accession numbers BX649209.1 and AP017625.1; M. liflandii, EU271968.1; M. marinum, EU271967.1) were obtained using PYANI v0.2.11 and a BLAST-based approach (ANIb) (15). The plasmid sequences were also aligned using Mauve v2.4.0 (16). We could not determine the mycolactone polyketide synthase genes, encoding the polyketide synthases required for mycolactone synthesis (17), on the plasmid from NJB1907-Z4 (Fig. 1).

These sequences will improve our understanding of the pathogenicity and evolution of this mycobacterium.

### Data availability.

The complete genome and plasmid sequences have been deposited in the DNA Data Bank of Japan (DDBJ) under the accession numbers AP026367 and AP026368, respectively. The raw sequencing reads are available in the DDBJ Sequence Read Archive under the accession numbers DRA013337 and DRA014909.
